# Functional Phenotypes of Peritoneal Macrophages Upon AMD3100 Treatment During Colitis-Associated Tumorigenesis

**DOI:** 10.3389/fmed.2022.840704

**Published:** 2022-05-09

**Authors:** Shuai Wu, Weiwei Luo, Xing Wu, Zhaohua Shen, Xiaoyan Wang

**Affiliations:** ^1^Department of Gastroenterology, The Third Xiangya Hospital, Central South University, Changsha, China; ^2^Key Laboratory of Non-resolving Inflammation and Cancer of the Hunan Province, The Third Xiangya Hospital, Central South University, Changsha, China

**Keywords:** colorectal cancer, tumorigenesis, CXCL12, AMD3100, peritoneal macrophages

## Abstract

CXCL12 and its receptor CXCR4 are independent prognostic factors in colorectal cancer. AMD3100 is the most frequently used FDA-approved antagonist that targets the CXCL12-CXCR4 axis in clinical trials. We aimed to explore the role of AMD3100 and its effect on peritoneal macrophages' functional phenotypes during colitis-associated tumorigenesis. We treated AMD3100 in a colitis-associated colon cancer mouse model and evaluated its effect on tumorigenesis. The phagocytosis activities of peritoneal macrophages were measured by flow cytometry. The proportions of macrophages and M1/M2 subpopulations were investigated by flow cytometry, ELISA, and immunochemistry. Serum levels of pro-inflammatory and anti-inflammatory cytokines were measured by LEGENDplex™ kits. Transwell assay and qRT-PCR were performed to investigate the direct effect of CXCL12 on macrophages *in vitro*. We demonstrated that AMD3100 treatment reduced the inflammatory damages in the colonic mucosal and ameliorated tumor development in experimental mice. We found that the phagocytosis activities of peritoneal macrophages fluctuated during colitis-associated tumorigenesis. The proportions of peritoneal macrophages and M1/M2 subpopulations, together with their metabolite and cytokines, changed dynamically in the process. Moreover, AMD3100 regulated the functional phenotypes of macrophages, including reducing the recruiting activity, promoting polarization to the M1 subpopulation, and reducing IL-12 and IL-23 levels in serum. Our study contributes to understanding dynamic changes of peritoneal macrophages upon AMD3100 treatment during tumorigenesis and sheds light on the potential therapeutic target of AMD3100 and peritoneal macrophages against colitis-associated colon cancer.

## Introduction

Colorectal cancer (CRC) is the third most commonly diagnosed cancer and the second leading cause of cancer-associated death worldwide (1). It is well established that chronic colitis predisposes individuals to colorectal tumorigenesis (2). Inflammatory bowel disease, such as Crohn's disease and ulcerative colitis, which features chronic recurrent intestinal inflammation of the intestinal tract, is a significant risk factor for patients developing colitis-associated colon cancer (CAC) (3, 4).

C-X-C motif chemokine ligand 12 (CXCL12) and its receptor C-X-C motif chemokine receptor 4 (CXCR4) have emerged as promising therapeutic approaches targeting tumor growth and metastasis (5). They were reported to promote cancer progression in several preclinical models, including CRC (6–11), and are independent prognostic factors for tumor differentiation, metastasis, and survival in CRC (12, 13). AMD3100 (Plerixafor) is currently the only FDA-approved small molecule CXCR4 antagonist and is the most frequently used drug targeting the CXCL12-CXCR4 axis in clinical trials (14). Research focusing on the potential application of CXCR4 antagonist against CRC in combination with or not with anti-PD-L1 antibodies is mounting (15, 16). Continuous administration of AMD3100 for 1 week in microsatellite stable CRC patients induced an integrated immune response, including intra-tumoral T and NK cell accumulation and activation (15). Combining anti-CXCL12 monotherapy with pembrolizumab triggered activation and clustering of T cells in the tumor core and led to long-term disease stabilization in patients with pretreated advanced metastatic colorectal cancer. Additionally, the efficacy of anti-CXCL12 is associated with the presence of the CXCL12-associated CD14^+^CD15^+^ cell population of the monocytic lineage in tissues (16).

CAC development is a multistep process encompassing chronic inflammation that accelerates tumorigenesis by the repeated cycles of epithelial wounding and repair and involves a complex interplay of infiltrated immune cells (17). Peritoneal macrophages (PMs), as the most abundant cell types in ascitic mononuclear cells in the peritoneal cavity, were demonstrated to mediate the peritoneal dissemination of CRC and gastric cancer (18, 19). However, unlike tumor-associated macrophages (TAMs) in the tumor microenvironment, which are known to regulate tumor proliferation and metastasis and are associated with the prognosis of numerous cancers (20), the role of PMs on cancer progression remains largely unknown. Rei et al. (21) found that PMs, mobilized by interleukin (IL)-17, are enriched in pro-inflammatory and proangiogenic mediators (*il1b, il6, vegfa, transforming growth factor-beta [tgfb], mif, cxcl1, and cxcl8*) and strongly and directly promote ovarian cancer proliferation. Wang et al. (22) found that during colitis-associated tumorigenesis, PMs gained aberrant expression of both the pro-inflammatory (*Il1b, Il6, Il12, tumor necrosis factor-alfa [Tnfa]*) and anti-inflammatory cytokines (*Il10, Tgfb*), which indicates its potential role in carcinoma development and metastasis.

Given macrophage's multifaceted role in physiological and pathological states, PMs may express different functional phenotypes in response to the complex microenvironmental signals in CRC. This study aimed to investigate the role of intraperitoneal treatment of AMD3100 and its effect on PMs' dynamic changes of subpopulations and functional phenotypes during colitis-associated tumorigenesis.

## Materials and Methods

### Mice

Eight to ten-week-old C57BL/6 mice were injected with 10 mg/kg azoxymethane (AOM, Sigma-Aldrich, St. Louis, MO, USA). All mice were given drinking water seven days later, adding 2.5% dextran sodium sulfate (DSS, M.P. Biomedicals, Solon, OH, USA; M.W., 36,000–50,000 Da) for the following seven consecutive days, followed by regular water for 2 weeks. This cycle was repeated three times then changed to regular water until the end of the experiment in the 18^th^ week (23). In the CAC+AMD group, mice were treated daily with AMD3100 (5 mg/kg, i.p) to interfere with the CXCL12/CXCR4 axis. In the CAC group, 1 × phosphate-buffered saline (PBS) was injected instead. All mice procedures were performed following institutional guidelines. The protocol was approved by the Animal Ethics Committee of Central South University for the protection of animals.

### Flow Cytometry

Peritoneal cells were aseptically isolated by peritoneal lavage with cold PBS containing 2% FBS and 1% penicillin-streptomycin. After centrifugation, cells were resuspended and washed with PBS. Cells were stained with the flow cytometry antibody anti-F4/80-PE-Cy7, anti-CD11b-FITC, anti-CD206-APC, and anti-CD16/32-PE (BD Biosciences, San Jose, CA, USA) for 30 min at 4°C. The cells were then washed and resuspended in PBS. The stained cells were analyzed using a flow cytometer (BD FACS CANTO II, BD Biosciences, San Diego, CA) and the FlowJo software (Tree Star, Ashland, OR).

### Measurement of iNOs and Arginase-1

The levels of iNOs and arginase-1 in mice colon tissue were measured by the NOS2 / iNOS (mouse) ELISA Kit (BioVision, Milpitas, CA, USA) and mouse ARG1/Arginase-1 ELISA kit (LifeSpan BioSciences, Seattle, Washington, USA) according to the manufacturer's instructions respectively.

### Hematoxylin and Eosin and Immunohistochemical Staining

Paraffin-embedded intestinal specimens from patients and experimental mice were stained with H&E for the microscopic examination. The sections were reviewed and scored based on the following criteria (24): inflammation (graded as 0 = normal, 1 = small leukocyte aggregates in mucosa and/or submucosa, 2 = coalescing mucosal and/or submucosal inflammation, 3 = coalescing mucosal inflammation with prominent multifocal submucosal extension +/- follicle formation, 4 = severe diffuse inflammation of mucosa, submucosa, and deeper layers), epithelial defects (0 = none, 1 = focally dilated glands and/or attenuated surface epithelium, decreased goblet cells, 2 = focally extensive gland dilation and/or surface epithelial attenuation, 3= erosions, 4 = ulceration), crypt atrophy (0 = none, 1 = <25%, 2 = 25–50%, 3 = 50–75%, 4 = >75%), and dysplasia/neoplasia (0 = normal, 1 = aberrant crypt foci, 2= polyploid hyperplasia/dysplasia, 3 = adenomatous and/or sessile hyperplasia/dysplasia, 3.5 = intramucosal carcinoma, 4 = invasive carcinoma), was added to extent of dysplasia/neoplasia (0 = none, 1 = <10% surface area, 2 = 10–25% surface area, 3 = 25–50% surface area, 4 = >50% surface area) for a final score.

IHC was performed on 4 μm-thick paraffin-embedded colorectal tissue sections from mice. The deparaffinized sections were incubated with a rabbit anti-iNOS antibody (Abcam, Cambridge, UK), a CD68 polyclonal antibody (Proteintech, Rosemont, IL, USA), or a Ly6G monoclonal antibody (Cell Signaling Technology, Danvers, MA, USA) at 4°C overnight followed by incubation with a biotinylated goat anti-rabbit or mouse IgG antibody (Vector Laboratories, Burlingame, CA, USA) for 30 min. The immunostained sections were observed under a microscope (OLYMPUS BX-51, Tokyo, Japan) and scored based on the immunoreactive score system (25): positive cells proportion score (graded as 0 = no positive cells, 1 = <10% of positive cells, 2 = 10–50% positive cells, and 3 = 51-80% positive cells, 4 = >80% positive cells) was multiply by staining intensity score (graded as 0 = no reaction, 1 = mild reaction, 2 = moderate reaction, and 3 = intense reaction) for a final score with 12 possible values.

### Measurement of Cytokines

In mice, serum cytokine levels (IL-10, IL-12, IL-17A, IL-23, TNF-α, and TGF-β) were estimated by LEGENDplex™ kits (Biolegend, San Diego, CA, USA) following the manufacturer's instructions.

### Transwell Migration Experiments

Transwell assays were used to examine cell motility. 1 × 10^5^ macrophages per well were seeded in the upper chamber in 100 μL of serum-free medium (24-well, 8.0 μm pore, Millipore). In the lower chamber, 600 μL of medium with 15% fetal bovine serum (FBS; Hyclone) was added, with or without 100 ng/ml recombinant CXCL12 (PeproTech). After incubated for 24 h at 37°C, cells at the upper surface of the membrane were removed. Cells on the lower surface of the membrane were fixed with 4% paraformaldehyde and stained with 0.1% crystal violet solution. Cell number was counted using an inverted fluorescence microscope.

### qRT-PCR

Total RNAs were isolated from cell lines using TRIzol reagent following the standard protocols and quantified using a Biospec-nano spectrophotometer (Life Science, Columbia, MD, USA). Total RNA was used to synthesize cDNA by reverse transcription with the SuperScript™ RT reagent Kit (Thermo Fisher Scientific, Waltham, USA) with a reaction system volume of 20 μL. The expression of the mRNAs was evaluated using SYBR green qRT-PCR (Takara Biotechnology Ltd., Dalian, China) following the standard protocol. Specific primer sequences used for PCR are as follows. Mouse IL-8: forward, 5′-CTCTGTGGTATCCAAGAATCAGTGA-3′, and reverse, 5′-TATTGCATCTGGCAACCCTACA-3′; mouse IL-10: forward, 5′-TGCAGGCTAACA CAGACACAG-3′, and reverse, 5′-AGCTGTGGGTTCTCATTCGC-3′; mouse IL-12: forward, 5′-GTCACAAAGGAGGCGAG GTT-3′, and reverse, 5′-CAGCAGGTG AAACGTCCAGA-3′; mouse IL-17A: forward, 5′-TACAACCGATCCACCTCACC-3′, and reverse, 5′-TTCACAGTGGTCCTTCCAGGT-3′; mouse IL-23: forward, 5′-AAAGGCAGCAGCTCAAGGAT-3′, and reverse, 5′-GTGCCTGGGGTGGTAGAT TT-3′; mouse TNF-α: forward, 5′-GGTGTCTGGCACACAGAAGAC-3′, and reverse, 5′-CTTA GCCCTGAGGTGTCTGG-3′; mouse TGF-β: forward, 5′- GGTTTGACCTAGGCGCTCA-3′, and reverse, 5′-CTGTCTGTGCTCACCCT CAC-3′; mouse IFN-γ: forward, 5′-TTCAGTTCCCAC GCCAATCT-3′, and reverse, 5′-TTGAAGAAG ATACCCAAATCAGTCT-3′.

### Phagocytic Activity

Macrophages were isolated as described above. After washing, 0.5 ml of 10% FBS-RPMI-1640 medium containing 25 μg of pHrodo Green Zymosan Bioparticles Conjugate for Phagocytosis (Invitrogen, Eugene, OR, USA) was added to each well and incubated for 2 h under a dark condition. After removing the culture medium, the cells were washed and collected in PBS, and phagocytic activity was measured on a flow cytometer.

### Statistics

All data were expressed as the means ± SEM of at least three independent experiments. Differences in the two groups were analyzed using the two-sided student's *t*-test or two-way ANOVA test by GraphPad Prism Software (La Jolla, CA, USA). A *p*-value of <0.05 was considered statistically significant.

## Results

### AMD3100 Inhibited AOM/DSS Induced CRC Tumorigenesis

**Figure 1:**
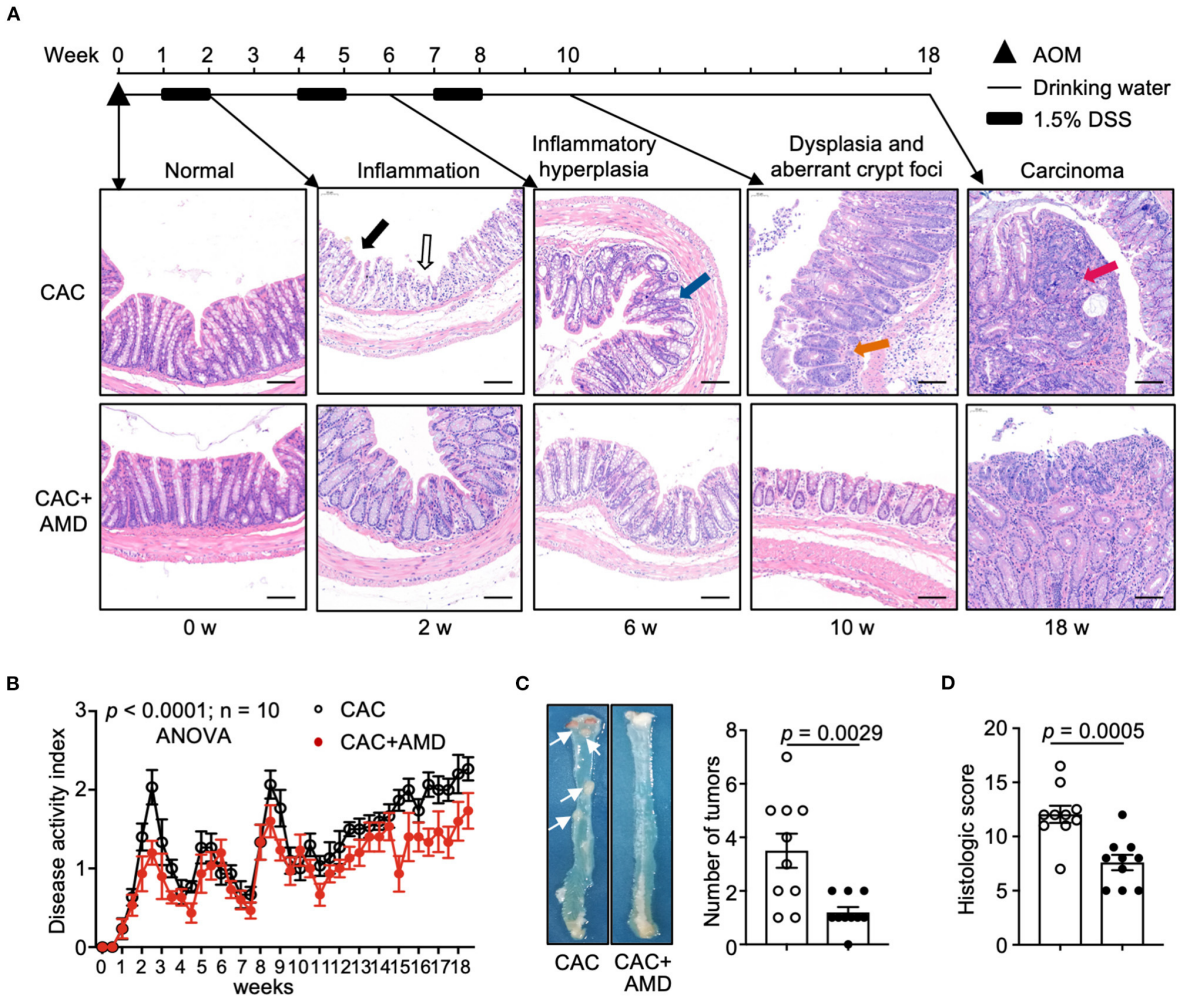
AMD3100 inhibited AOM/DSS-induced CRC tumorigenesis. **(A)** Schematic representation of the experimental design and representative images of morphological observation of the colon of mice injected with PBS (CAC) or with AMD3100 (CAC + AMD) at indicated time intervals (in untreated mice and in mice from the end of 2^nd^, 6, and 18^th^ weeks, respectively) after azoxymethane (AOM)/ dextran sodium sulfate (DSS) treatment (scale bar = 100 nm, magnification 100×). The solid black arrow indicates colonic mucosa with a damaged epithelial barrier. The black hollow arrow indicates erosion and crypt abscesses. The Blue arrow indicates inflammatory hyperplasia in the colonic tissue with crypt distortion. The orange arrow indicates dysplasia and aberrant crypt foci. The red arrow indicates an overt carcinoma polyp in the lumen of the proximal colon. **(B)** Changes of disease activity index (DAI) during the treatment of AOM/DSS in mice from two groups (*n* = 10). DAI = (weight loss score + stool characteristic score + hematochezia score)/3. **(C)** Representative gross colon morphology and the numbers of tumors were measured at the 18^th^ week of AOM/DSS administration (*n* = 10). The white arrow indicates the overt carcinoma polyps. **(D)** Histopathology scores of mice in two groups in the 18^th^ week of treatment (*n* = 10). Data are shown as Mean ± SEM **(B)** or Mean ± SD. Statistical analysis was carried out using Two-way ANOVA with Geisser-Greenhouse correction and multiple unpaired *t*-tests.

To investigate the role of AMD3100 in CAC development, we modeled the colitis-associated tumorigenesis process in mice with carcinogen AOM and pro-inflammatory agent DSS. Pathological analyses revealed a “normal–inflammation-low-grade dysplasia–high-grade dysplasia–carcinoma” sequential process in the colon as the natural disease process in humans (23), which is represented in the colonic mucosa as inflammation by week 2, inflammatory hyperplasia by week 6, dysplasia and aberrant crypt foci by week 10, and carcinoma at weeks 18 (Figure 1A).

After induction of colitis with DSS in the 2^nd^ week, we observed severe epithelial barrier destruction in the colonic mucosa from CAC mice. The goblet cells were lost and replaced by infiltrated inflammatory cells and crypt abscesses (indicated by black arrows). Part of the epithelial layer of the intestinal mucosa was shed and fused into large ulcers. However, colonic tissues from AMD3100-treated mice largely retained the integration of the epithelial barrier and formed scattered ulcers. Six weeks after induction, atypical hyperplasia appeared in the colorectal mucosa, accompanied by inflammatory cells infiltrating. The arrangement of glands was slightly disordered, where the glandular hyperplasia cells were of different sizes and shapes, with hyperchromatic nuclei, increased nucleocytoplasmic ratio, and disordered polarity (indicated by blue arrows). In mice treated with AMD3100, however, we observed scattered glandular hyperplasia cells, but the structures of glands and crypts remain integrated. At the end of the 10^th^ week after modeling, high-grade intraepithelial neoplasia appeared in the intestinal mucosa of CAC mice. The gland cavities, which consisted of atypia cells, were of different sizes and irregular shapes and surrounded by inflammatory cells (indicated by orange arrows). At the end of the 18^th^ week, all CAC mice developed colorectal cancer, which protruded intestinal mucosa to form adenocarcinoma polyps, where the glandular structure of intestinal mucosa disappeared. Some polyps are fused into large adenocarcinomas with inflammatory cells infiltrating around the cancerous tissue. In the colon of AMD3100 treated mice, we observed milder epithelial barrier damage and less dysplasia crypt at the end of the 10^th^ week and more diminutive carcinoma polyps in the 18^th^ week than in CAC mice (Figure 1A).

The overall trends of DAI scores, used to evaluate the severity of colitis, increased following the ongoing experiment in both groups. In AMD3100-treated mice, the mean DAI scores were lower than those of untreated littermates, which indicated milder symptoms, such as less weight loss, milder diarrhea, and alleviated bleeding in the intestine (Figure 1B). In addition, AMD3100 treatment reduced the number of tumor polyps in colonic tissues (Figure 1C) and attenuated colonic damages and relative histologic scores in the 18^th^ week (Figure 1D). These findings suggested that AMD3100 treatment attenuated the inflammatory damage in the colonic tissues and inhibited the AOM/DSS-induced CRC tumorigenesis.

### The Phagocytic Function of PMs Changed During Colitis-Associated Tumorigenesis

**Figure 2:**
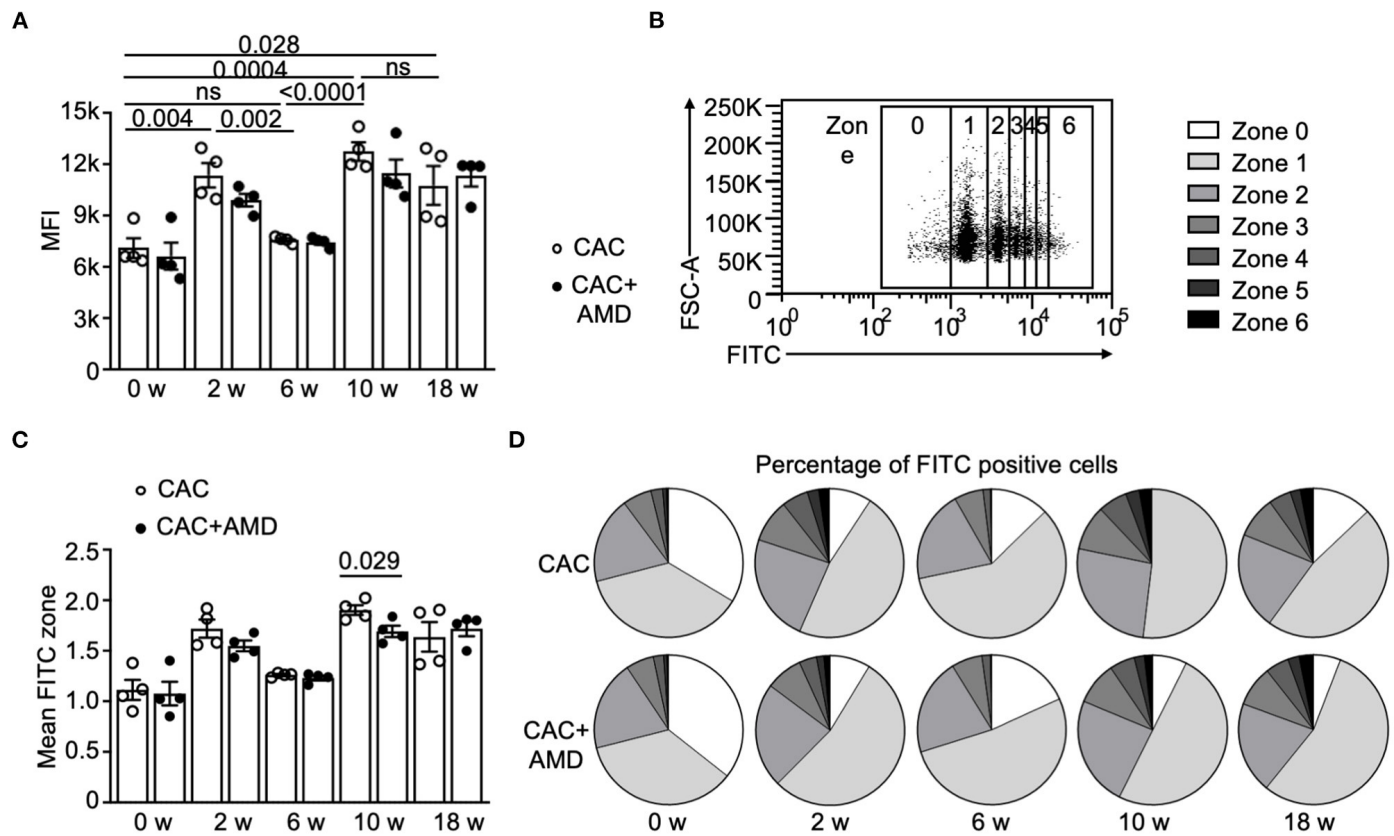
The phagocytic function of PMs changed during colitis-associated tumorigenesis. **(A)** The flow cytometer measured the phagocytic activity of peritoneal macrophages (PMs) from two groups. Mean fluorescence intensity (MFI) of the pHrodo Green Zymosan Bioparticles phagocyted by macrophages was calculated and shown (*n* = 4). **(B)** The gating strategy for macrophages with increased phagocytic functions was marked as 0-6 depending on the MFI value on FITC. **(C)** Mean FITC zone of PMs from two groups (*n* = 4). **(D)** Percentage of PMs with different phagocytic functions from two groups in untreated mice and in mice from the 2^nd^, 6, 10, and 18^th^-weeks post-AOM/DSS induction (*n* = 4). Individual data are shown as Mean ± SD. Statistical analysis was carried out using unpaired *t*-tests.

One essential function of macrophages is phagocytosing foreign substances, such as pathogens, apoptotic cells, and other abnormal invaders, including tumor cells. The phagocytosis and subsequent immune recognition of tumor cells have been considered a bridge of innate and adaptive immunity to trigger anti-tumor responses and provide a new avenue for developing cancer immunotherapies (26). However, little is known about the phagocytic function of PMs in AOM/DSS-induced CAC. In this study, we found that the phagocytosis activity of macrophages, which was quantified by the mean fluorescence intensity (MFI), increased in the 2^nd^ week, decreased to baseline in the 6^th^ week, and increased again in the 10 and 18^th^ weeks. The differences between them were significant (Figure 2A).

To further quantify macrophage phagocytosis, we classified the macrophages into seven (zero to six) zones according to their FITC fluorescence values (Figure 2B) and analyzed the proportion of macrophages in each zone. At the beginning and in the 6^th^ week, around 70% of macrophages belong to zone zero and one, but the proportions decreased below 60% in the 2^nd^, 10, and 18^th^ weeks (Figures 2C,D). The overall trend of the mean FITC zone is similar to the changes in MFI, except that AMD3100 treatment decreased the mean FITC zone in PMs at the 10^th^ week (Figures 2C,D), indicating that AMD3100 influenced the phagocytosis activity of PMs at specific microenvironment.

### AMD3100 Inhibited PM Recruiting and Regulated the Proportions of the M1/M2 Subpopulation

Macrophages in tumor tissues are the most abundant innate immune cells in the tumor microenvironment (27). Macrophages are capable of polarizing toward a spectrum of phenotypes, including classically pro-inflammatory and anti-tumor M1 subpopulation and the alternative pro-healing and pro-tumor M2 phenotype (28).

**Figure 3:**
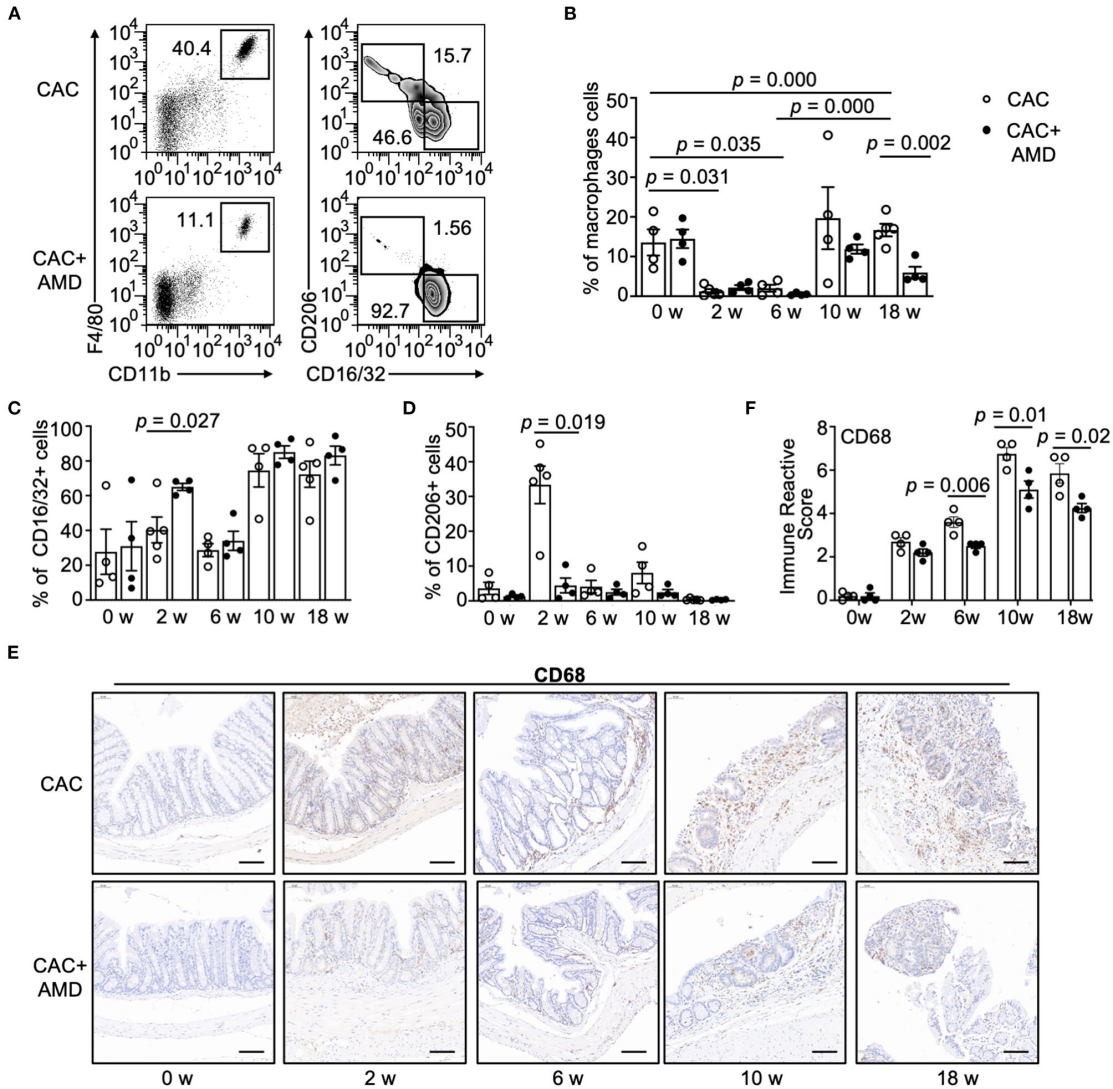
AMD3100 inhibited PM recruiting and regulated the proportions of the M1/M2 subpopulation. **(A)** Flow cytometric characterization of macrophages in representative samples of peritoneal lavage cells in mice from the 10^th^ week post-AOM/DSS induction. Cells were identified as follows: macrophages = CD11b^+^F4/80^+^, M1 = CD11b^+^F4/80^+^CD16/32^+^CD206^−^, and M2 = CD11b^+^ F4/80^+^CD16/32^−^CD206^+^. **(B)** Percentage of macrophages in peritoneal lavage cells in two groups in untreated mice and mice from the 2^nd^, 6, 10 and 18^th^-weeks post-AOM/DSS induction (*n* ≥ 4). **(C,D)** Percentage of CD16/32^+^CD206^−^ M1 type and CD16/32^−^CD206^+^ M2 type macrophages in peritoneal macrophages (PMs) from two groups in the 0^th^, 2^nd^, 6, and 18^th^-weeks post-AOM/DSS induction (*n* ≥ 4). **(E,F)** Representative images of immunohistochemical staining for CD68 in colonic mucosa in mice from each group at 0, 2, 6, 10, and 18 weeks after AOM/DSS treatment and quantified immune reactive scores (scale bar = 50 nm, magnification 200×). Individual data are shown as Mean ± SD. Statistical analysis was carried out using unpaired *t*-tests.

In our study, two-parameter histograms F4/80+CD11b+ were recognized as macrophages and further divided by CD16/32 and CD206 as specific markers for M1 and M2, respectively (Figure 3A). In the CAC mice model, we found a significant decrease in PMs' percentage in the 2^nd^ and 6^th^ weeks, and the proportion increased in the 10 and 18^th^ weeks. The overall trend of the PMs' percentage in the AMD3100-treated mice was lower than that in the CAC mice, and the difference was significant in the 18^th^ week (Figure 3B). In addition, the proportion of the M2 subpopulation peaked at the second week but decreased to the baseline afterward, whereas the M1 subpopulation largely remained stable at the baseline in the 2^nd^ and 6^th^ weeks but increased in the 10 and 18^th^ weeks in the CAC mice. However, in AMD3100-treated mice, the proportion of M1 cells was significantly higher than that in the CAC group in the 2^nd^ week. The increasing trend in the AMD3100 group was shown at other time points after AOM/DSS induction, but the differences were not significant (Figure 3C). Meanwhile, the proportion of M2 cells was relatively low in the colonic tissues of AMD3100-treated mice, especially in the 2^nd^ week when the difference was significant (Figure 3D). Furthermore, we detected the CD68+ macrophages in colonic tissues using IHC and quantified them by immune reactive score. We found that the amounts of CD68+ macrophages significantly increased in the colonic lamina propria from mice in two groups in the 2^nd^, 6, 10, and 18^th^ weeks. In addition, in the colon of AMD3100-treated mice, the amounts of CD68+ macrophages were significantly lower than that in the CAC mice (Figures 3E,F). These data suggested that AMD3100 inhibited PMs recruiting during CAC progress and regulated the proportions of the M1/M2 subpopulation at the inflammation stage.

### AMD3100 Regulated the Levels of iNOs and Arginase-1 in Colonic Tissues During CAC

**Figure 4:**
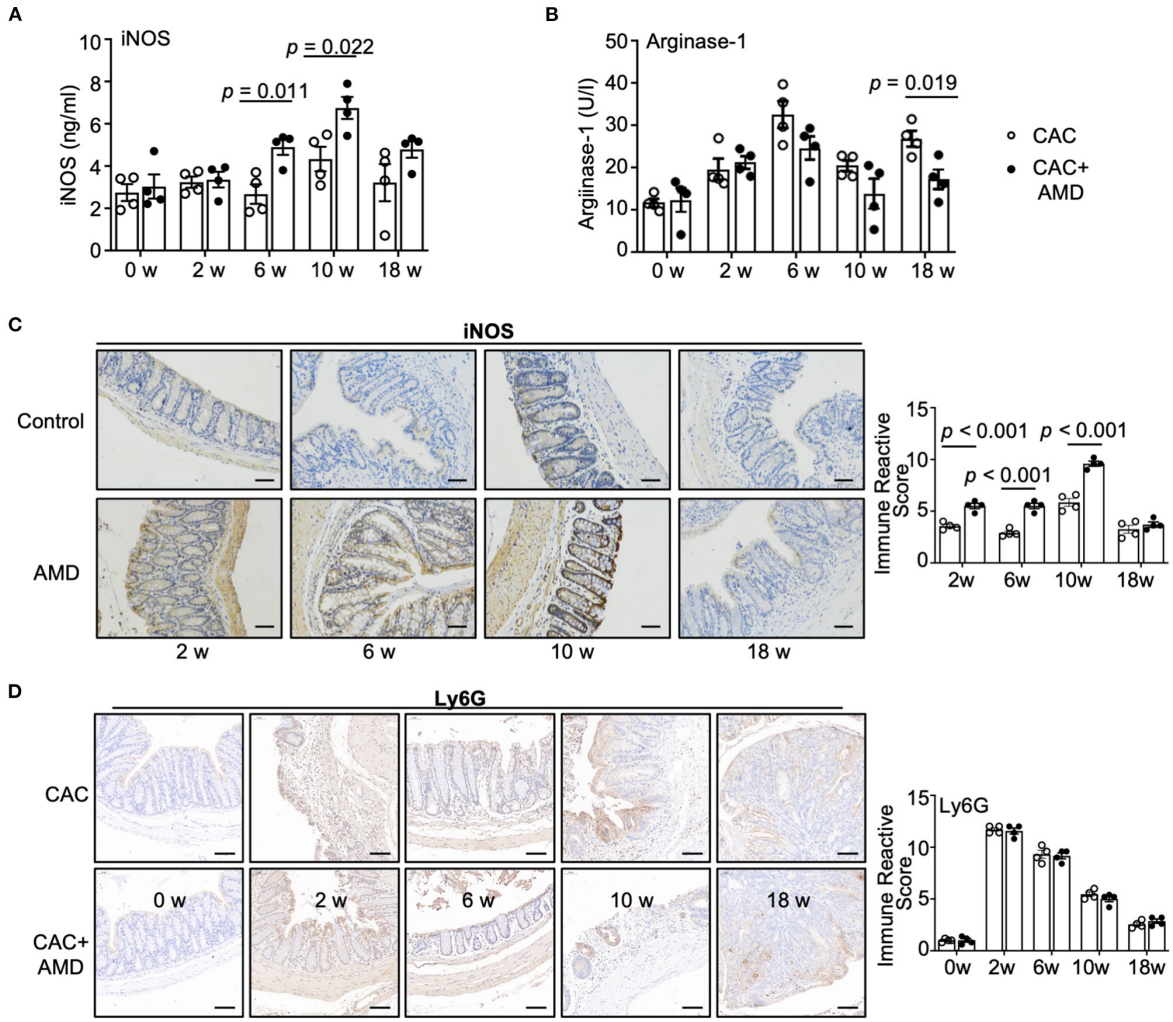
AMD3100 regulated the levels of iNOS and arginase-1 in colonic tissues during CAC. **(A)** The expression of inducible nitric oxide synthase (iNOS) in colonic tissues was measured by ELISA (*n* = 4). **(B)** The expression of arginase-1 expressions in colonic tissues was measured by ELISA (*n* = 4). **(C)** The expression of iNOS was assessed by immunohistochemical staining and quantified by immune reactive scores (scale bar = 50 nm, magnification 200×). **(D)** The expression of Ly6G was assessed by immunohistochemical staining and quantified by immune reactive scores (scale bar = 50 nm, magnification 200×). Individual data are shown as Mean ± SD. Statistical analysis was carried out using unpaired *t*-tests.

M1 macrophages have a potent ability to metabolize arginine to nitric oxide (NO) via the inducible nitric oxide synthase (iNOS), whereas M2 macrophages preferentially metabolize arginine to ornithine via arginase-1 (29). To further investigate the M1/M2 subpopulations, we performed ELISA and IHC staining to evaluate iNOS and arginase-1, the specific markers of pro-inflammatory and pro-healing phenotypes of macrophages. We found that the levels of iNOS in colonic tissues were increased at the 10^th^ week in the CAC mice. In addition, the levels of iNOS were higher in AMD3100-treated mice than in untreated mice in the 6 and 10^th^ weeks (Figure 4A). Meanwhile, the levels of arginase-1 increased in both groups in the 2^nd^, 6, 10, and 18^th^ weeks. Moreover, compared to the CAC mice, AMD3100 treatment decreased the levels of arginase-1 in the 6, 10^th^, and especially in the 18^th^ week, where the difference was significant (Figure 4B). In addition, the IHC staining of iNOS revealed that AMD3100 treatment increased the levels of iNOS in the colonic tissues in the 2^nd^, 6, and 10^th^ weeks after modeling (Figure 4C). Furthermore, considering that neutrophils were another source of iNOS (30), we detected the Ly6G+ neutrophils in colonic tissues using IHC. Upon the AOM/DSS treatment, we found significantly increased Ly6G+ cells resident in the mucosa in the 2^nd^, 6, 10, and 18 weeks, especially in the 2^nd^ week when it reached the highest level. However, the immune reactive scores between the CAC and AMD3100-treated groups did not differ (Figure 4D). Hence, AMD3100 treatment regulated the levels of iNOS and arginase-1 but not the amount of Ly6G+ neutrophils in colonic tissues during CAC.

### AMD3100 Reduced Serum Levels of IL-12 and IL-23 at the Late Stage of CAC

**Figure 5:**
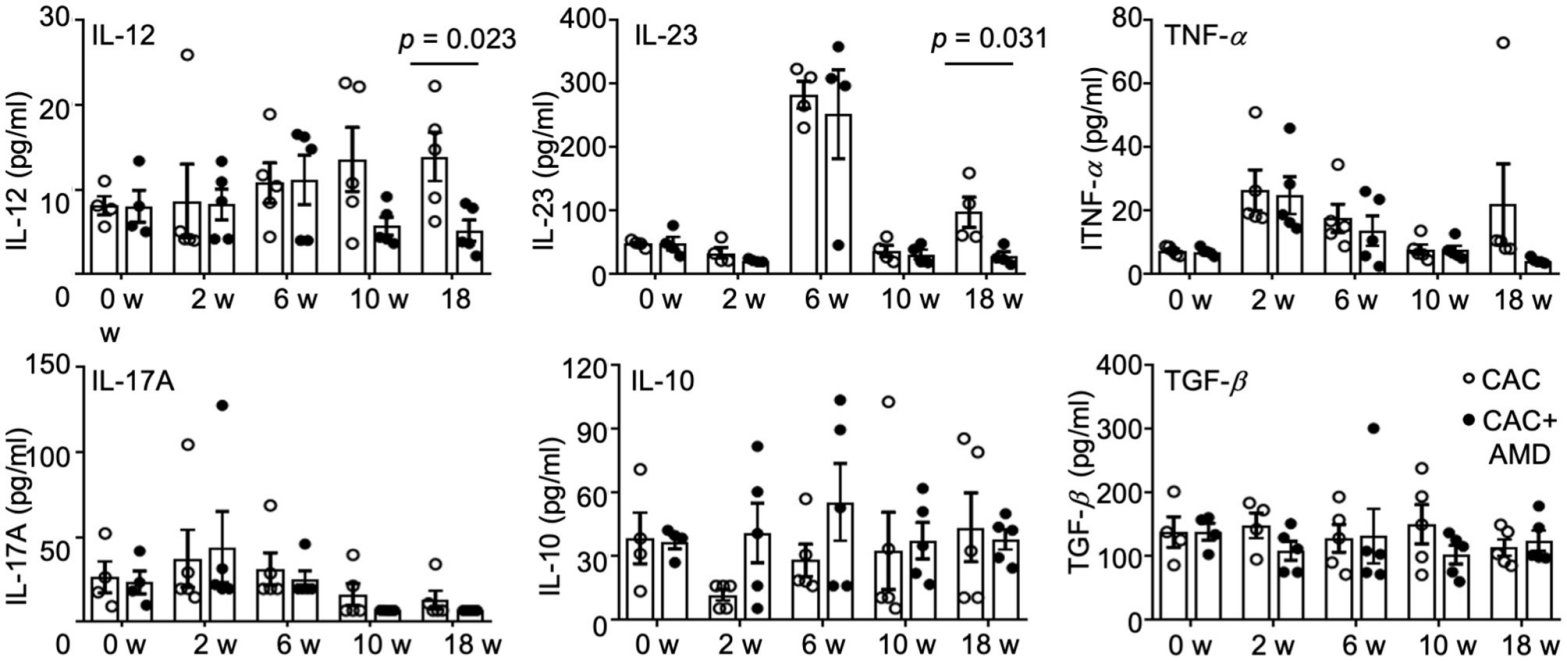
AMD3100 regulated serum levels of cytokines. The levels of IL-12, IL23, TNF-α, IL17A, IL-10, and TGF-β were measured in serum from control and AMD mice in the 0^th^, 2^nd^, 6, 10, and 18^th^ weeks post-AOM/DSS treatment by LEGENDplex™ kits (*n* ≥ 4). Individual data are shown as Mean ± SD. Statistical analysis was carried out using unpaired *t*-tests.

During cancer development, chronic inflammation was induced and functioned as a crucial promoter, along with changes in the cellular microenvironment that favor tumor formation, in which many cell types interact via secreted factors (31). In the present study, we measured the IL-10, IL-12, IL-17A, IL-23, TNF-α, and TGF-β cytokines in mice serum at different time points using LEGENDplex™ kits. We found that the level of pro-inflammatory cytokine IL-12 slightly increased during cancer development in the 6, 10, and 18^th^ weeks. The level of another member of the IL-12 family, IL-23, increased dramatically in the 6^th^ week but decreased to the baseline afterward. Levels of TNF-α and IL-17A increased in the 2^nd^ and 6^th^ weeks and decreased to the baselines in the 10 and 18^th^ weeks. Meanwhile, the anti-inflammatory cytokines IL-10 and TGF-β remain relatively stable as the baselines throughout the treatment. AMD3100 treatment reduced the level of IL-12 in the 10^th^ week and especially in the 18^th^ week. In addition, the level of IL-23 in the AMD3100-treated mice significantly reduced compared to that in CAC mice in the 18^th^ week (Figure 5). Collectively, these results imply that AMD3100 treatment regulated the serum levels of IL-12 and IL-23 at the late stage of CAC.

### CXCL12 Directly Regulated Macrophages' Functions

**Figure 6:**
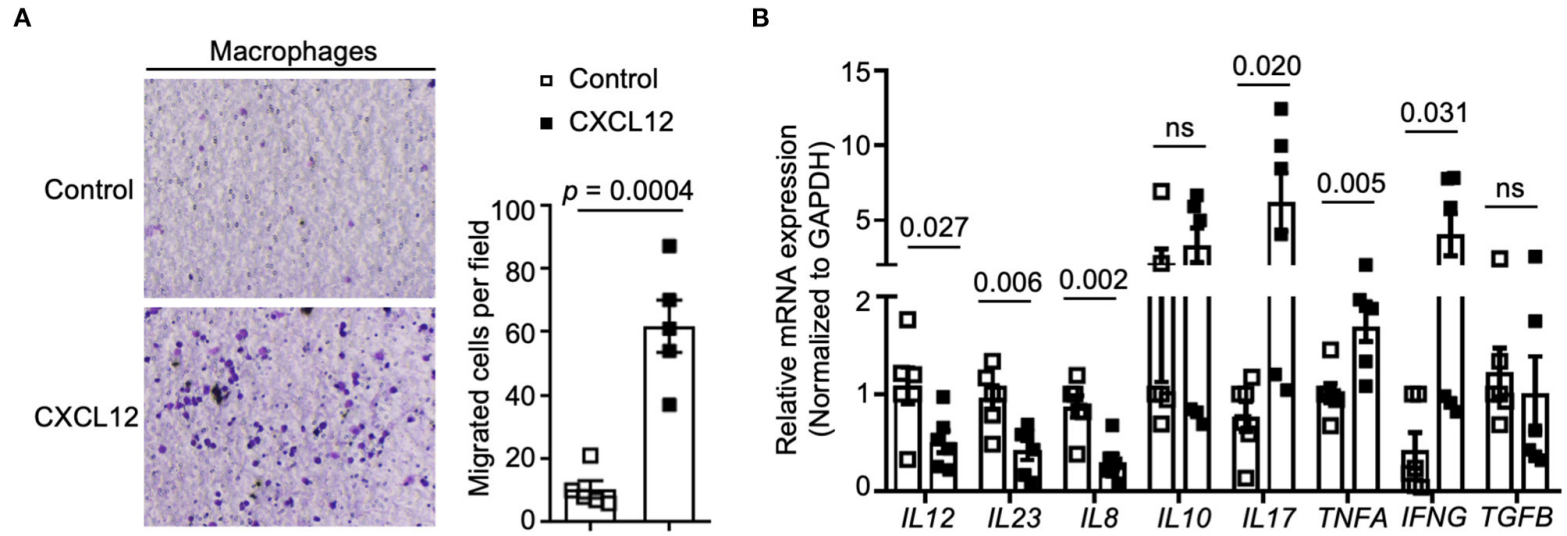
CXCL12 regulated macrophages' recruitment and cytokines expression. **(A)** Transwell assays were performed to measure cell recruitment capacity, and the numbers of migrated cells were counted (*right panel*). Macrophages were stimulated with CXCL12 (100 ng/ml) or not (*n* = 5). **(B)** Relative expression of *IL-12, IL-23, IL-8, IL-10, IL17A, TNF-*α*, IFN-*γ, and *TGF-*β were analyzed by qRT-PCR. Individual data are shown as Mean ± SD. Statistical analysis was carried out using unpaired *t*-tests.

AMD3100 is the specific antagonist blocking the CXCL12/CXCR4 axis. To explore the direct effect of AMD3100 on macrophages, we used CXCL12 to stimulate macrophages *in vitro*. We found that upon CXCL12 stimulation, the number of migrated macrophages were increased markedly compared to that in the untreated group, which indicated an increased recruitment ability of macrophages (Figure 6A). Moreover, after CXCL12 stimulation, macrophages express lower mRNA levels of *IL-12, IL-23*, and *IL-8* and higher *IL-17, TNF-*α, and *IFN-g*. Meanwhile, *IL-10* and *TGF-*β did not show many differences between groups upon treatment (Figure 6B).

## Discussion

The CXCL12/CXCR4 axis is crucial signaling for tumor cells cross-talking with the tumor microenvironment that paves a path for tumorigenesis (32). AMD3100 is a specific CXCR4 antagonist used frequently in clinical trials for solid tumors. In the current study, we showed that blocking CXCL12/CXCR4 axis by specific antagonist AMD3100 reduced the inflammatory damage in the colonic mucosal and ameliorated the development of CAC in experimental mice. We found that the phagocytosis activity of PMs fluctuated during the CAC process. In addition, the proportions of PMs and M1/M2 subpopulations, together with their metabolite and cytokines, changed dynamically during colitis-associated tumorigenesis. Nevertheless, AMD3100 regulated the phagocytosis activity of PMs, the proportion of the M1/M2 subpopulation, and the level of its specific metabolites and cytokines. Experiments in cells confirmed the effect of CXCL12 on macrophages *in vitro*.

CXCL12 and CXCR4 are independent prognostic factors for tumor differentiation, metastasis, and CRC survival (12, 13). Considering the high conservation of the CXCL12/CXCR4 axis across diverse species, studies of CXCR4 inhibitors in mouse and cell lines have the potential to translate into human clinical studies quickly. AMD3100, in combination with or not with anti-PD-L1 antibodies, has been applied in immunotherapeutic against CRC, pancreatic cancer, hepatocellular carcinoma, and breast cancer (15, 16, 33, 34). Biasci et al. found that continuous administration of AMD3100 in microsatellite stable CRC patients induced an integrated immune response, including intra-tumoral T and NK cell accumulation and activation, and decreased serum levels of ctDNA and circulating CXCL8. Even though it did not reveal clinical remissions due to the short period, it provides early indications of the possibility of an early anti-cancer effect mediated by CXCR4 inhibition (15). Another study found that applicated combination therapy of anti-CXCL12 monotherapy with pembrolizumab increased T-lymphocyte infiltration and improved immunotherapeutic effect in patients with pretreated advanced metastatic CRC, which also revealed that the efficacy of anti-CXCL12 is associated with the presence of CXCL12-associated CD14^+^CD15^+^ cell population of the monocytic lineage (16).

Previously, we demonstrated that restoring CXCR4 reversed the inhibition effects of miR-126 on promoting the migration, proliferation, and invasion of colon cancer cells (35). Other studies showed that blocking CXCR4 attenuates colonic damage in the murine colitis model (36, 37), indicating its critical role in the intestinal inflammatory response and CRC progression. We consistently found in the current study that AMD3100 markedly ameliorated the colonic barrier damage, inflammatory infiltration, and colitis symptoms at the inflammation stage and reduced the histologic scores and tumor numbers at the late stage. Most adverse events of long-term administration of AMD3100 in patients were mild, transient, and resolved without treatment, including abdominal pain, fatigue, headaches, tachycardia, injection site reactions, nausea, and bloating (15, 16, 38). In this study, we did observe sensitive abdomen in a few mice treated with AMD3100. However, after repeated cycles of DSS treatment, most mice performed abdominal symptoms, including abdominal pain, diarrhea, and loose stool, and the adverse events in the AMD3100-treated group in contrast to the non-treated group were hard to tell.

Under pathologic conditions such as cancer, the volume of fluid in the peritoneal cavity increases, and its biochemical composition changes, which often correlates with poor prognosis in patients (39). Ascites are reservoirs of a complex mixture of resident peritoneal cells, constitutive or inducible products, and sometimes invading pathogens and cancer cells (40). PMs are the most abundant cell types in ascitic mononuclear cells in the peritoneal cavity and the first line to defense invaders. Although the general role of macrophages in the colonic tissues in CRC has been well demonstrated, given the multifaceted role that macrophage has in physiological and pathological states, the functional phenotype of PMs may reveal some diversity.

Our research demonstrated the dynamic changes of PMs under the treatment of AMD3100 during the AOM/DSS-induced CRC progress. At the acute inflammation stage (the 2^nd^ and 6^th^ weeks), we observed increased CD68+ macrophages infiltrating the inflamed colonic lamina propria, which lasted until the end of the experiment. In the meantime, consistent with previous findings, we found a significant increase of Ly6G+ neutrophils in the damaged mucosa in the 2^nd^ and 6^th^weeks after modeling, and the mucosal infiltration of neutrophils declined after the acute inflammation stage (41, 42). As the most abundant circulating leukocyte and the first responders to sites of infection and acute tissue damage (43), the massively accumulated neutrophils in the injured sites might explain the decline proportions of PMs in the 2^nd^ and 6^th^ weeks and the increased proportion in the 10 and 18^th^ weeks when the number of neutrophils gradually declined. Still, as the neutrophil outnumbers other leukocytes by a wide margin, it might shrink the differential alteration in macrophages at the 2^nd^ and 6^th^ weeks.

CXCL12/CXCR4 was reported to drive the accumulation of tumor-associated macrophages in the tumor environment or egress to draining lymphatics (44, 45). In contrast, another study found that CXCL12 stimulation of CXCR4 inhibited the directed migration of human immune cells mediated by chemokine receptors, such as CCR2/CCL2, for the migration of monocytes and macrophages (15). Moreover, in certain pathological states, like stroke and diabetes, blocking the CXCL12/CXCR4 axis by AMD3100 treatment led to elevated populations of activated macrophages in the ischemic cortex or skin wounds (46, 47). Consistent with the previous studies (8, 44), we found a decreased amount of CD68+ macrophages and proportions of PMs in the AMD3100-treated mice in the current study.

Although PMs did not express detectable cell surface levels of CXCR4, they did respond to stimulations of CXCL12, and its expression increased during acute inflammatory peritonitis (48, 49). The increased expression of CXCR4 in macrophages under inflammation stimuli explained the increased amount of CD68+ macrophages after modeling and increased proportions of PMs at the 10 and 18^th^ weeks when the intestinal inflammation response aggravated alongside CRC development. In addition, consistent with that, recruitment of neutrophils to the tumor location was also regulated by chemokines like CXCL12 (50). CXCL12 has been reported to synergize with IL-8 to chemoattract neutrophils or independently induce neutrophil recruitment (51, 52). In contrast, as an HIV antagonist, phase-I clinical studies indicated that AMD3100 causes a rapid rise in circulating leukocytes, including neutrophils (53). In mice, AMD3100 treatment was shown to mobilize lymphocytes, monocytes, and neutrophils from primary immune organs to secondary immune organs, peripheral tissues, and blood (54, 55). However, despite that AMD3100-induced neutrophil mobilization to blood, it did not reduce neutrophil trafficking to inflamed peripheral tissues (55, 56). Consistent with these studies, we did not find differences between CAC and AMD3100-treated groups.

Emerging studies have demonstrated that increased tumor-associated macrophage phagocytic activity improved the T-cell mediated adaptive immune responses and promoted the detection and clearance of malignant cells (26). The combination of anti-CXCR4 and anti-PD-1 immunotherapy mainly focus on T cell responses but not innate anti-cancer immunity (15, 16, 33, 34). Our research found PMs' increased phagocytic activity in the 2^nd^ week after the first treatment cycle of DSS when acute inflammatory responses were triggered. In the 6^th^ week, however, the phagocytic activity decreased to the baseline level when the critical time of formatting inflammatory hyperplasia and dysplasia might indirectly contribute to tumor development. Surprisingly, the phagocytic activity increased and remained at a stable high level at the 10 and 18^th^ weeks, and AMD3100 treatment slightly decreased it. Nevertheless, possibly due to T cell exhaustion or tumor immune escape at a later stage of tumorigenesis (57), we did not observe the influence of phagocytosis on developing CRC.

At the extremes of their phenotypic continuum, macrophages can be classified into two subtypes: pro-inflammatory M1 macrophages produce type I pro-inflammatory cytokines such as TNF-a and IL-12 and cytotoxic substances like iNOS. Conversely, anti-inflammatory M2 macrophages produce type II cytokines like IL-10 and arginase-1, facilitating collagen deposition and promoting tissue repair. High levels of M2 macrophage infiltration are associated with a poor prognosis in CRC patients (58). Therefore, reprogramming from the M2 phenotype to the M1 phenotype is effective in cancer therapy, and the role of peritoneal M1/M2 macrophages upon AMD3100 treatment during CRC tumorigenesis needs to be evaluated.

Increased macrophages, particularly M2 subtype macrophages, correlated with metastasis and poor CRC prognosis (59, 60). The present study found that the proportion of M2 macrophages markedly increased in the 2^nd^ week but quickly decreased and remained relatively stable afterward. In contrast, the subpopulation of M1 macrophages remained at a stable baseline during the inflammation and dysplasia process (in the 2^nd^ and 6^th^ weeks) but increased in aberrant crypt foci and carcinoma (10 and 18^th^ weeks). Additionally, in the 2^nd^ week, treatment of AMD3100 significantly increased the proportion of M1 macrophages and decreased the M2 subpopulation in the peritoneal cavity.

It has been shown that the chemokine CXCL12 regulated the differentiation of monocyte-to-macrophage by downregulating the expression of the transcription factor RUNX3 and induced macrophages to differentiate to an M2 phenotype characterized by higher surface expression of CD163 (61). In addition, M2 macrophages expressed a higher level of CXCR4 than M1 macrophages (62), which might also contribute to the changes in the proportion of peritoneal M1/M2 macrophages.

As a critical member of the mononuclear phagocyte network, the M1 and M2 macrophages' subpopulation is also distinguished by phagocytic activity. Chatterjee et al. (63) believe that M1 macrophages have high phagocytic activity than the M2 subpopulation, whereas Kapellos et al. (64) hold the opposite opinion. Our study found that AMD3100 promoted PMs skewing to the M1 subpopulation but slightly decreased its phagocytic activity, which unveiled a fragment of the diversity phenotype of PMs.

NO is a free radical produced by a family of NOSs by the oxidation of L-arginine. iNOs, as an inducible enzyme of pro-inflammatory M1 macrophage, also existed in other cell types such as neutrophils, vascular smooth muscle cells, microglia, and neurons (65). In the current study, we found that the levels of iNOS in colonic tissues were increased at the 10^th^ week while the levels of arginase-1 increased at the end of 2^nd^ and 6^th^ weeks but decreased slightly at the 10 and 18^th^ weeks. In addition, AMD3100 treatment increased the levels of iNOS and decreased the levels of arginase-1. As the amounts of Ly6G+ neutrophils in the two groups did not show a difference, the dynamic changes might be related to the changes in M1/M2 macrophage subpopulations.

The exact role of iNOS and NO in cancer remains obscure, as it has both tumor promoter and suppressor effects depending on the local environment. It has been reported that NO inhibited tumor cell proliferation, differentiation, and metastatic spread (66). In a chemical colitis model and AOM/DSS induced CAC mouse model, Stettner et al. (67) demonstrated that NO induction of endogenous NO production by enterocytes improved epithelial integrity and alleviation of colitis and of inflammation-associated colon cancer. On the contrary, Erdman et al. (68) found that in *Helicobacter hepaticus*-infected Rag2-deficient mice, administration of an iNOS inhibitor prevented NO production and abrogated epithelial pathology, and inhibited the onset of colon cancer. Another study reported that endogenous NO plays a vital role in defining the stemness properties of colon cancer stem cells, and either a specific iNOS inhibitor or a genetic knock-down of iNOS significantly reduced the tumourigenic capacities of colon cancer stem cells (69).

Cytokines are crucial mediators of inflammation and immunity and have diverse and pleiotropic roles at different CRC progression stages (70). We detected several representative cytokines in the mice serum and found that anti-inflammatory cytokines IL-10 and TGF-β remained stable during the CRC development, whereas the pro-inflammatory cytokines changed in different patterns. Serum IL-17A, IL-23, and TNF-α increased at the inflammation and dysplasia stage, and the IL-23 dramatically elevated in the 6^th^ week particularly. Another pro-inflammatory cytokine, IL-12, was raised in the 10 and 18^th^ week while carcinoma was developing. In addition, the AMD3100 decreased serum IL-12 and IL-23 in the 18^th^ week. However, *in vitro* CXCL12 stimulation decreased the mRNA level of *IL12* and *IL23* in macrophages. Additionally, CXCL12 treatment *in vitro* increased *IL-17, TNFA*, and *TGFB* and reduced *IL8*. This inconsistency indicates the complex orchestrated response of immune cells other than PMs in the inflammation-tumor microenvironment during the CRC progression under the influence of AMD3100.

The “normal-inflammation-dysplasia-dysplasia–carcinoma” sequential pathological transition is a complex process that takes a long time and involves numerous cells and features. In the CAC mouse model, we observed a transition from chronic inflammation to dysplasia around 6 weeks after modeling and transited colonic mucosa with dysplasia and aberrant crypt foci at the 10th week. In the current study, the administrated of AMD3100 was performed from the beginning of the CAC model. However, considering the dynamic changes in the microenvironment in the colonic tissues at different CAC stages, it is not sufficient to administrate AMD3100 from the beginning of modeling. In future studies, we will try to start administering AMD3100 at several time points, like in the 6 or 10^th^ weeks, which will help us better understand the exact role of AMD3100 in colitis-associated tumorigenesis in different stages.

The present study contributes to understanding the role of AMD3100 and its effect on the dynamic changes of PMs' functional phenotypes. However, there are still disadvantages to this study. First, besides M1 and M2 functional milieu, PMs can also be divided into small PMs and large PMs according to their morphology (71). Further studies are needed to investigate the different functions of these two subsets in CRC development. Second, the combined effect of immune cells and cytokines drives the CRC progression. For instance, dendritic cells play a crucial role in presenting tumor antigens and inducing T cell-primed anti-tumor response (72). T cells exert anti-tumor function through releasing cytotoxic molecules, anti-tumor cytokines and directly inducing tumor cell death (73). Even though less explored, B cells are an independent predictor of a favorable clinic outcome of CRC (74, 75). Therefore, the interplay between PMs, dendritic cells, T cells, and B cells in the tumor microenvironment during CRC progression, including the combined effects of timing, calls for further attention.

## Conclusion

In conclusion, the current study showed that AMD3100 treatment reduced the inflammatory damages in the colonic mucosal and ameliorated colitis-associated colon cancer development in experimental mice. We demonstrated the dynamic profile of peritoneal macrophages during the tumorigenesis process. In addition, we found that AMD3100 regulated the functional phenotypes of PMs, including phagocytosis activity, recruitment, polarization, and the expression of cytokines. Our study contributes to understanding dynamic changes of peritoneal macrophages upon AMD3100 treatment during tumorigenesis and sheds light on the potential therapeutic target of AMD3100/macrophages in treating CAC.

## Data Availability Statement

The original contributions presented in the study are included in the article/supplementary material, further inquiries can be directed to the corresponding author/s.

## Ethics Statement

The animal study was reviewed and approved by the Animal Ethics Committee of Central South University for the protection of animals (No. 2018-S092, December 2018).

## Author Contributions

SW performed most of the experiments and analysis and drafting of the manuscript. WL performed IHC detection in colon tissues. XWu and ZS participated in the analysis and discussion of results. XWa supervised the project and revised the manuscript. All authors contributed to the article and approved the submitted version.

## Funding

This work was funded by grants from the National Natural Science Foundation of China (Nos. 81272736, 81670504, and 81472287).

## Conflict of Interest

The authors declare that the research was conducted in the absence of any commercial or financial relationships that could be construed as a potential conflict of interest.

## Publisher's Note

All claims expressed in this article are solely those of the authors and do not necessarily represent those of their affiliated organizations, or those of the publisher, the editors and the reviewers. Any product that may be evaluated in this article, or claim that may be made by its manufacturer, is not guaranteed or endorsed by the publisher.
